# Cancer-released exosomal circular RNA circ_0008717 promotes cell tumorigenicity through microRNA-1287-5p/P21-activated kinase 2 (PAK2) axis in non-small cell lung cancer

**DOI:** 10.1080/21655979.2022.2056822

**Published:** 2022-03-25

**Authors:** Huimin Wang, Zhiqin Tang, Jihui Duan, Chunlei Zhou, Ke Xu, Hong Mu

**Affiliations:** a Department of Clinical Laboratory, Tianjin First Center Hospital, Tianjin, Hebei, China; b Tianjin Key Laboratory of Lung Cancer Metastasis and Tumor Microenviroment, Tianjin Lung Cancer Institute, Tianjin Medical University General Hospital, Tianjin, Hebei, China

**Keywords:** Exosomes, circ_0008717, miR-1287-5p, PAK2, NSCLC

## Abstract

Circular RNA (circRNA) circ_0008717 has been revealed to promote cell carcinogenesis in non-small cell lung cancer (NSCLC). Exosomal circRNA packaged into exosomes has been defined as a potential diagnostic and therapeutic biomarker of cancers. However, little attention is focused on the role of circRNAs within exosomes in NSCLC. Exosomes were isolated by ultracentrifugation method and qualified by nanoparticle tracking analysis and Western blot. Levels of circ_0008717, microRNA (miR)-1287-5p, and P21-activated kinase 2 (PAK2) were detected using qRT-PCR and western blot. The interaction between miR-1287-5p and circ_0008717 or PAK2 was investigated. The phenotypes of NSCLC cells with circ_0008717 downregulation were tested. Circ_0008717 was highly expressed in NSCLC. Functionally, circ_0008717 deficiency suppressed cell malignant phenotypes in NSCLC *in vitro* and in nude mice. Circ_0008717 sponged miR-1287-5p to elevate PAK2, a downstream target of miR-1287-5p. Silencing of miR-1287-5p blocked the antitumor effects of circ_0008717 knockdown in NSCLC cells. Besides, miR-1287-5p repressed cell oncogenic behaviors in NSCLC by targeting PAK2. Besides that, we confirmed that circ_0008717 was incorporated into exosomes in NSCLC cells. Circ_0008717 knockdown inhibited NSCLC tumorigenesis via miR-1287-5p/PAK2 axis, and the extracellular circulating circ_0008717 was transferred through incorporation in exosomes.

## GRAPHICAL ABSTRACT

**Figure:**
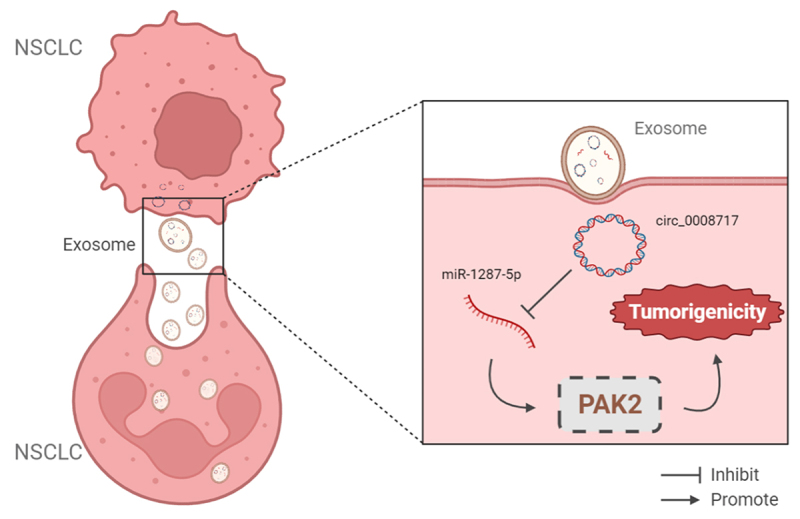

## 1 Introduction

Non-small cell lung cancer (NSCLC) is one of the deadliest cancers worldwide with a five-year survival rate less than 17.1% [1,2]. Thus, it is of utmost importance to probe the mechanisms of NSCLC carcinogenesis for the development of advanced medicines to improve clinical outcomes in NSCLC patients. Currently, the extracellular vesicle exosomes have been identified to mediate tumor communication [3] and can transfer and exchange their cargo, like protein, lipids, or noncoding RNAs, into tumor cells and the extracellular microenvironment [4,5], thus affecting the development of many types of cancers. Exosomes have been identified that may be potentially implicated in the tumorigenesis of malignancy and act as potential clinical biomarkers for the therapy and diagnosis of cancers via the transfer of proteins and circular RNAs (circRNAs) [6,7].

Recently, non-coding RNAs (ncRNAs) have been proposed to be the potential modulators of cancer cell carcinogenesis, metabolism, and drug resistance [8–10]. CircRNAs are defined as highly conserved ncRNAs possessing covalently closed-loop structures. Thus, they can be resistant to the degradation of exonuclease [11,12]. Importantly, circRNAs are identified to play significant roles in diverse cellular responses, developmental and disease processes [13,14]. Thus, circRNAs may be promising candidates for future therapeutic interventions in cancers [15]. Circ_0008717 (circABCB10) is a novel identified circRNA. Fu et al. showed that circABCB10 was elevated in hepatocellular carcinoma (HCC) and predicted poor survival. What is more, knockdown of circABCB10 suppressed cell proliferative and invasive abilities in HCC by down-regulating HMG20A via miR-670-3p [16]. Zhou et al. uncovered that high circ_0008717 expression was directly linked to low survival in osteosarcoma, and circ_0008717 induced the progression of malignant cell phenotypes to promote osteosarcoma development through binding to the miR-203/Bmi-1 axis [17]. Additionally, it was also demonstrated that circ_0008717 was elevated in NSCLC, and promoted cancer progression via miR-1252/FOXR2 axis [18]. In any case, whether exosomal circ_0008717 serves as a therapeutic biomarker for NSCLC still needs further study.

Therefore, in this study, we aimed to explore circ_0008717 derived from exosome on mechanisms underlying the progression of NSCLC. Exosomal circ_0008717 expression in NSCLC serum and cells were measured, and then we further explored the action and molecular mechanisms of circ_0008717 in NSCLC progression using *in vitro* and *in vivo* assays.

## 2 Materials and methods

### 2.1 Tissues samples and serum collection

Tumor tissues, adjacent normal tissues, and blood samples from 48 NSCLC patients at Tianjin First Center Hospital were acquired. The blood samples collected from 48 healthy controls were used as control. The supernatant serum was obtained by centrifugation and was immediately preserved at −80°C until use. Written informed consent had been provided by all recruiters and the Ethics Committee of Tianjin First Center Hospital allowed this research.

### 2.2 Cell culture

Human NSCLC cell lines (A549 and H1299) and bronchial epithelial cells (BEAS-2B) were bought from Chuan Qiu Biotechnology (Shanghai, China) and cultivated in the RPMI-1640 medium (Invitrogen, Carlsbad, CA, USA) plus 1% penicillin/streptomycin and 10% FBS at 37°C with 5% CO_2_.

### 2.3 Exosome (exo) isolation

The isolation of exosomes was performed using differential ultracentrifugation and 0.22 μm filtration as previously described [19]. Purified exosomes were collected and used immediately, or were resuspended in PBS to observe the morphology of exosomes using transmission electron microscopy (TEM) (JEOL, Akishima, Japan), or to investigate the size and the distribution using nanoparticle tracking analysis (NTA) with the Zeta View 8.04.04 SP2 7 software.

### 2.4 Western blot analysis

The RIPA lysate harboring protease inhibitor (Beyotime, Beijing, China) was employed to extract proteins from exosomes, cells, or tumor tissues. Then immunoblotting was carried with primary antibodies (Abcam, Cambridge, MA, USA) included CD63 (1:2000, ab68418), CD81 (1:5000, ab109201), P21-activated kinase 2 (PAK2) (1:3000, ab76293), Bcl-2 (1:2000, ab182858), GADPH (ab181602, 1:10000), and Bax (1:1000, ab32503). Immunoreactive bands were observed employing the ECL system (Beyotime) and quantified with Image Lab software.

### 2.5 qRT-PCR

The extraction of whole-RNA from exosome pellets, cells, or tissues was performed by TRIzol reagent (Invitrogen) according to the standard procedure. Then Prime Script RT Master Mix and SYBR Premix Ex Taq (Qiagen, Valencia, CA, USA) were utilized for cDNA generation and qRT-PCR analysis, respectively. The relative expression was assessed using 2^−ΔΔCt^ method with the internal references of GADPH and U6. The sequence of primers was listed: circ_0008717: F 5’-GCCTTTCCATTCCGTCAGGA-3’, R 5’-ACCACGCTCAAAACAAAGGTG-3’; miR-1287-5p: F 5’-AGCTGGATCAGTGGTTCGAG-3’, R 5’-CAGTGCAGGGTCCGAGGTAT-3’; PAK2: F 5’-TCTTCCTCCCCCAGGGTTG-3’, R 5’-AATCGAGCCCACTGTTCTGG-3’; GAPDH: F 5’-GAAGGTGAAGGTCGGAGTC-3’, R 5’-GAAGATGGTGATGGGATTTC-3’; U6: F 5’-GCTTCGGCAGCACATATACTAAAAT-3’, and R 5’- CGCTTCACGAATTTGCGTGTCAT-3’.

### 2.6 Cell transfection

The siRNA targeting circ_0008717 covalent closed junction (si-circ_0008717#1, si-circ_0008717#2), pcDNA3.1 PAK2 overexpression vector (pc-PAK2), and corresponding negative control (NC) (si-NC, pc-NC), miR-1287-5p mimic, inhibitor, and the NC (miRNA NC, or inhibitor NC), as well as lentiviral particles carrying either a scrambled sequence (sh-NC) or circ_0008717-specific shRNA (sh-circ_0008717) were provided by Invitrogen (USA). All cell transfection was performed using lipofectamine 2000 (Invitrogen) according to the manufacturer’s instruction.

### 2.7 MTT assay

Transfected cells were interacted with MTT reagent (Beyotime) fixed by fresh medium (200 µL) in a 96-well plate. After 4 h post-reaction, the formed precipitation per well was dissolved by incubating with 200 µL dimethyl sulfoxide (DMSO; Beyotime). The absorbance was assayed using a spectrophotometer at 490 nm.

### 2.8 Colony formation assay

Transfected cells were seeded into 6-well plates at 500 cells per well with fresh medium for 14 days incubation. Then the culture was terminated, and colonies were counted after 0.1% crystal violet staining.

### 2.9 Transwell assay

The transfected cells were diluted to 5 × 10^4^/mL with complete medium and then planted in the top of transwell chambers without (migration assay) or with (invasion assay) the matrigel membranes, and 500 μL medium plus 10% FBS was filled into the lower chambers. Following 24 h hatch, migrated and invaded cells in five random fields were selected to count using a microscope after being stained.

### 2.10 Cell cycle analysis

Transfected cells were digested by trypsin to obtain single-cell suspensions. After being fixed by 75% ethanol for 4 h, cells were mixed with propidium iodide (PI, Beyotime). The distribution of cells was examined via the FACS Calibur flow cytometry.

### 2.11 Tube formation assay

A 48-well plate was filled with 75 μL Matrigel (Beyotime). After solidification, HUVECs were suspended in the indicated cell conditioned medium and incubated for another 5 h. Then images were obtained using a bright-field microscope, and the number of branches was assessed.

### 2.12 Cell apoptosis assay

Transfected cells were resuspended with a binding buffer and then stained with Annexin V-FITC and PI (Beyotime) following the standard protocol, and apoptotic cells were detected by flow cytometry.

### 2.13 Dual-luciferase reporter assay

The sequences of circ_0008717 or PAK2 3'UTR possessing binding sites in miR-1287-5p or mutated sequences in the binding region were cloned into the pGL3 luciferase reporter vectors (Promega, Shanghai, China). Then the recombinant luciferase reporter vectors and pRL-TK Renilla vector with indicated miRNAs were co-transfected into NSCLC cells, and luciferase activities were tested after 48 h of transfection.

### 2.14 RNA pull-down assay

NSCLC cells were infected with biotin-labeled circ_0008717 probe (circ_0008717 probe) and a control probe (Oligo probe) provided by GenePharma (Shanghai, China), followed by lysing and proceeding to incubate with streptavidin-coated magnetic beads. Finally, the abundance of RNAs was measured by qRT-PCR analysis.

### 2.15 RNA immunoprecipitation (RIP) assay

NSCLC cells were lysed by RIP buffer, followed by interacting with magnetic beads (Millipore, Billerica, MA, USA) conjugated with normal mouse IgG or human anti-Ago2, and proceeding to digest the protein using Proteinase K. Finally, the immunoprecipitated RNA was extracted for qRT-PCR.

### 2.16 *Xenograft experiments* in vivo

A549 cells transfected with lentivirus mediated sh-circ_0008717 or sh-NC were injected into the nude mice subcutaneously (4–6 weeks old). The tumor size was determined weekly. Mice were euthanized at day 28, and tumors were isolated, weighed, and used for the detection of circ_0008717, miR-1287-5p, and PAK2 using western blot or qRT-PCR. Also, tumors were fixed in formalin for immunohistochemistry (IHC) with the PAK2 antibody (1:250, ab76293, Abcam). All experiments were performed in line with the guidelines approved by the Ethics Committee of Tianjin First Center Hospital.

### 2.17 Statistical analysis

Data were presented as the mean ± standard deviation (SD). One-way analysis of variance (ANOVA) or Student’s *t*-test was utilized to determine the statistical differences between different groups with GraphPad Prism 7 software. *P* values <0.05 suggested statistical significance.

## 3 Results

### 3.1 Circ_0008717 is packaged into exosomes in NSCLC

**Figure 1.**
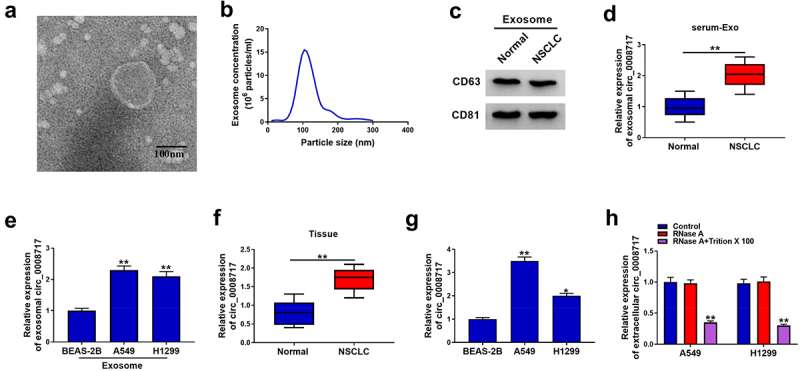
**Circ_0008717 is packaged into exosomes of NSCLC**. (a) Exosomes from serum were analyzed under transmission electron microscopy; (b) Nanoparticle tracking analysis was used to measure exosome particle size and concentration; (c) Western blot for CD63 and CD81 expression in exosomes; (d) Levels of circ_0008717 expression in exosomes from serum in NSCLC and normal; N = 48 per group; (e) QRT-PCR detection of circ_0008717 expression in exosomes from bronchial epithelial cells BEAS-2B and NSCLC cells; (f–g) Circ_0008717 expression was determined by qRT-PCR in NSCLC and normal tissues, as well as BEAS-2B and NSCLC cells. (h) Circ_0008717 expression was detected by qRT-PCR in A549 and H1299 incubating with RNase A alone or in combination with Triton X-100. All experiments repeated three times. *P < 0.05 and ** *P* < 0.01.

To investigate exosome-based mechanisms underlying the progression of NSCLC, we extracted and characterized exosomes from serum. A round shape with double-layer membrane structure was observed in the vesicles (Figure 1a). Then NTA further showed that the size distribution of vesicles was about 100 nm (Figure 1b). In addition, CD63 and CD81, the exosomal markers, were detectable in the isolated vesicles (Figure 1c). All these data indicated the successful isolation of exosomes. Subsequently, the expression of circ_0008717 was discovered to be higher in exosomes isolated from NSCLC patients and cells than those of healthy controls and normal BEAS-2B cells (Figure 1(d,e)). Additionally, a significant elevation of circ_0008717 was shown in NSCLC tissues and cells (Figure 1(f,g)). Additionally, qRT-PCR analysis displayed that RNase A treatment alone failed to affect circ_0008717 expression, while the level of circ_0008717 was overtly declined in the RNase A and Triton X 100 group (Figure 1h), revealing the package of circ_0008717 in exosomes. Moreover, the association between circ_0008717 expression and pathological characteristics was analyzed, and results exhibited that high circ_0008717 expression was linked with tumor size and advanced TNM stages (Table 1). In all, these results indicated that circ_0008717 was packaged into exosomes, and exosomal circ_0008717 was tightly associated with NSCLC progression.

**Table 1.** Correlation between circ_0008717 expression and clinical clinicopathological parameters of NSCLC patients

Parameter	Case	Circ_0008717 expression		*P* value
		Low (n = 24)	High (n = 24)	
Age (years)				0.562
≤ 60	22	12	10	
> 60	26	12	14	
Gender				0.149
Female	25	15	10	
Male	23	9	14	
Tumor size				0.0004**
≤ 5 cm	20	16	4	
> 5 cm	28	8	20	
TNM stages				0.0192*
I–II	20	14	6	
III–IV	28	10	18	

TNM, tumor-node-metastasis; *P < 0.05 and ** *P* < 0.01.

### 3.2 Circ_0008717 knockdown suppresses cell growth and mobility in NSCLC

**Figure 2.**
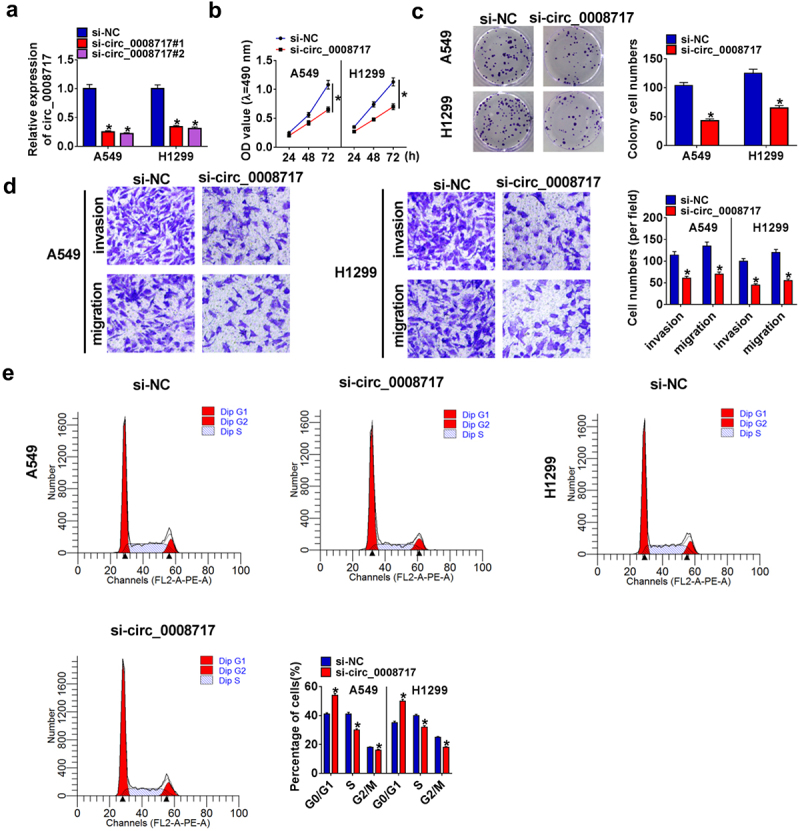
**Circ_0008717 knockdown suppresses cell growth and mobility in NSCLC**. Following assigned transfection, (a) qRT-PCR analysis of circ_0008717 expression in A549 and H1299 cells; (b–c) The cell proliferation of A549 and H1299 cells were determined by MTT (b) and colony formation assay (c); (d) Transwell assay (100 ×) was used to detect cell invasion and migration; (e) Cell cycle was analyzed by Flow cytometry. Error bars stand for the mean ± SD of three independent measurements. *P < 0.05.

Following the transfection of si-NC or si-circ_0008717 (si-circ_0008717#1 and si-circ_0008717#2), we found that si-circ_0008717 notably reduced circ_0008717 expression (Figure 2a), and then si-circ_0008717#2 (also shown si-circ_0008717) was chosen for subsequent experiments. Functionally, knockdown of circ_0008717 repressed the proliferation of A549 and H1299 cells (Figure 2(b,c)). Besides, transwell assay showed that si-circ_0008717 suppressed the invasion and migration of A549 and H1299 cells (Figure 2d). Meanwhile, we discovered that A549 and H1299 cells in the G0/G1 phase were accumulated upon circ_0008717 silencing but decreased in the S phase after circ_0008717 decrease, indicating cell cycle arrest (Figure 2e).

### 3.3 Circ_0008717 knockdown suppresses angiogenesis and induces apoptosis in NSCLC

**Figure 3.**
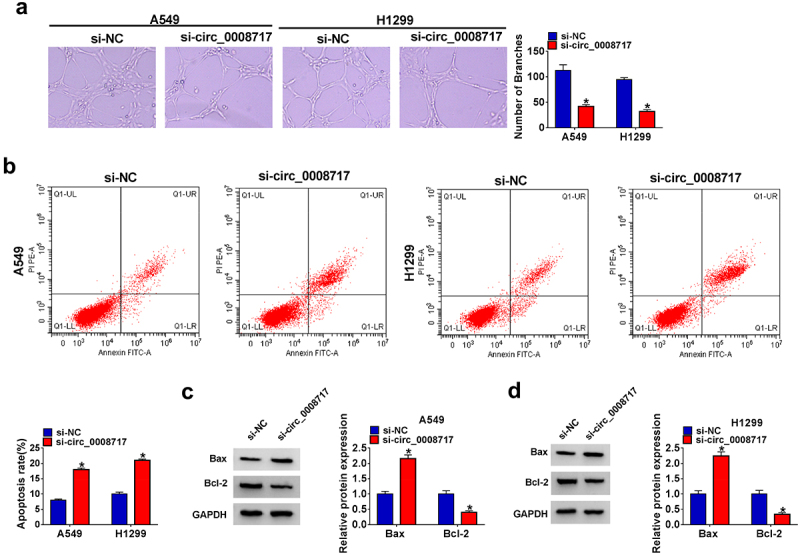
**Circ_0008717 knockdown suppresses angiogenesis and induces apoptosis in NSCLC**. A549 and H1299 cells were transfected with circ_0008717 siRNA (si-circ_0008717) and siRNA NC (si-NC); (a) Tube formation assay for HUVECs with the conditioned medium of indicated cells. (b) Flow cytometry of cell apoptosis rate. (c–d) Protein levels of Bax and Bcl-2 protein in cells were assayed by western blot. Error bars stand for the mean ± SD of three independent measurements. *P < 0.05.

Moreover, the number of formed branches of HUVECs was reduced in the si-circ_0008717 group (Figure 3a). In addition, apoptotic A549 and H1299 cells were promoted by si-circ_0008717 (Figure 3b), accompanied by the decrease of Bcl-2 and increase of Bax proteins (Figure 3(c,d)). Taken together, circ_0008717 knockdown inhibited NSCLC progression by suppressing cell oncogenic behaviors.

### 3.4 MiR-1287-5p is a target of circ_0008717 in NSCLC cells

**Figure 4.**
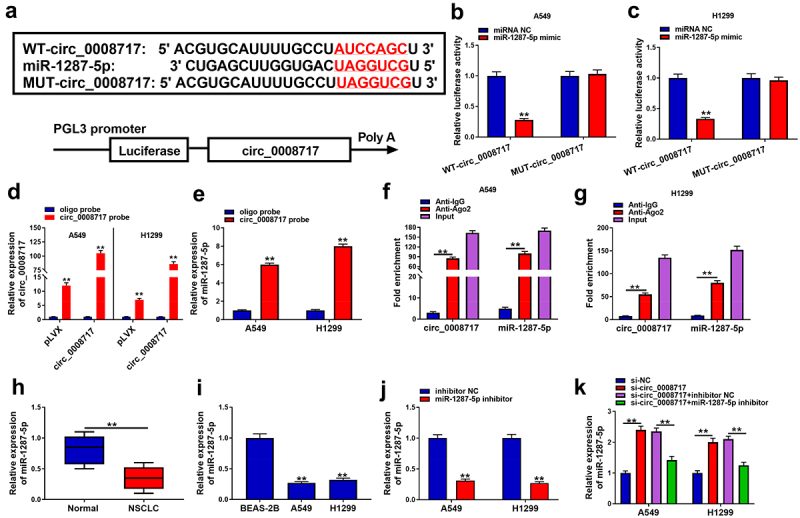
**MiR-1287-5p is a target of circ_0008717 in NSCLC cells**. (a) The binding sites of miR-1287-5p on circ_0008717. (b–g) The binding between miR-1287-5p and circ_0008717 was validated using dual-luciferase reporter, pull-down and RIP assays. (h,i) qRT-PCR analysis of miR-1287-5p expression in NSCLC tissues and normal adjacent tissues, as well as in normal BEAS-2B cells and NSCLC cells. (j) The interference efficiency of miR-1287-5p inhibitor or inhibitor NC was detected by qRT-PCR in A549 and H1299 cells. (k) qRT-PCR analysis was conducted to investigate the effects of circ_0008717 on miR-1287-5p expression in A549 and H1299 cells. Error bars stand for the mean ± SD of three independent measurements. *P < 0.05 and ** *P* < 0.01.

To elucidate whether circ_0008717 acts as a miRNA sponge to regulate NSCLC progression, we searched the database of CircInteractome (https://circinteractome.nia.nih.gov/index.html). Circ_0008717 possesses the binding sites of miR-1287-5p (Figure 4a). MiR-1287-5p mimic dramatically suppressed the luciferase activity of WT-circ_0008717 rather than MUT-circ_0008717 (Figure 4(b,c)). Meanwhile, we designed a biotinylated-circ_0008717 probe to conduct a pull-down assay. The enrichment of circ_0008717 in by circ_0008717 probe confirmed the pull-down efficiency (Figure 4d). Then qRT-PCR analysis displayed the enrichment of miR-1287-5p in circ_0008717 probe compared with the oligo probe (Figure 4e). Moreover, relative to the control Anti-IgG group, we also found circ_0008717 and miR-1287-5p were significantly enriched in the Ago2 overexpression group (Figure 4(f,g)). MiR-1287-5p expression was discovered to be decreased in NSCLC tissues and cell lines (Figure 4(h,i)). Also, the effect of circ_0008717 on miR-1287-5p expression was investigated. First, we detected that miR-1287-5p inhibitor predominantly suppressed miR-1287-5p expression relative to inhibitor NC in A549 and H1299 cells (Figure 4j). Next, qRT-PCR analysis also indicated that circ_0008717 silence notably increased miR-1287-5p expression level in A549 and H1299 cells, which was reversed by miR-1287-5p inhibitor (Figure 4k). Collectively, circ_0008717 directly bound to miR-1287-5p.

### 3.5 Silencing of miR-1287-5p attenuates the antitumor effects mediated by si-circ_0008717 in NSCLC cells

**Figure 5.**
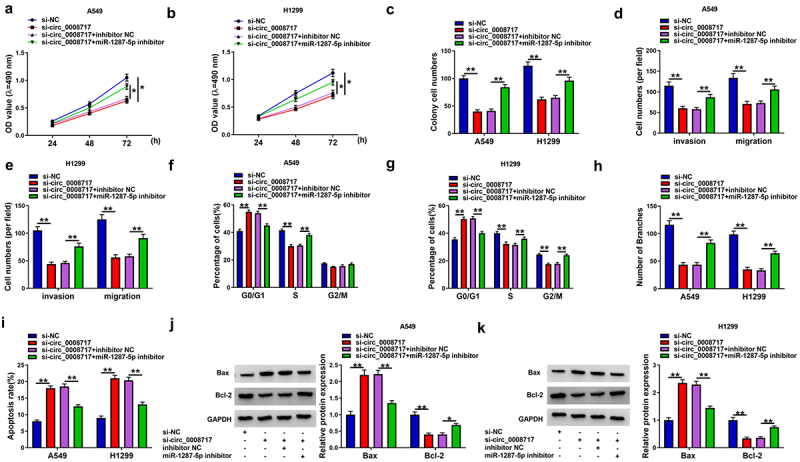
**Silencing of miR-1287-5p attenuates the antitumor effects mediated by si-circ_0008717 in NSCLC cells**. After indicated transfection, (a–c) The proliferation analysis of cells with MTT assay and colony formation assay. (d,e) Transwell assay for cell invasion and migration. (f,g) Flow cytometry for cell cycle analysis. (h) Tube formation in HUVECs by conditioned medium from indicated cells. (i) Flow cytometry of cell apoptosis rate. (j,k) Bax and Bcl-2 protein levels in cells were tested by western blot. Error bars stand for the mean ± SD of three independent measurements. *P < 0.05 and ** *P* < 0.01.

We then explored whether the action of circ_0008717 was mediated by miR-1287-5p using rescue assay. We proved that miR-1287-5p inhibition abolished si-circ_0008717-mediated repression of the proliferative (Figure 5(a–c)), migratory, and invasive abilities (Figure 5(d,e)) in A549 and H1299 cells. Besides, miR-1287-5p inhibitor abrogated circ_0008717 silencing-mediated cell cycle arrest (Figure 5(f,g)), decrease of branch number (Figure 5h), and promotion of cell apoptosis (Figure 5(i–k)) in A549 and H1299 cells. Thus, we showed that circ_0008717 promoted NSCLC progression by binding to miR-1287-5p.

### 3.6 PAK2 is a target of miR-1287-5p in NSCLC cells

**Figure 6.**
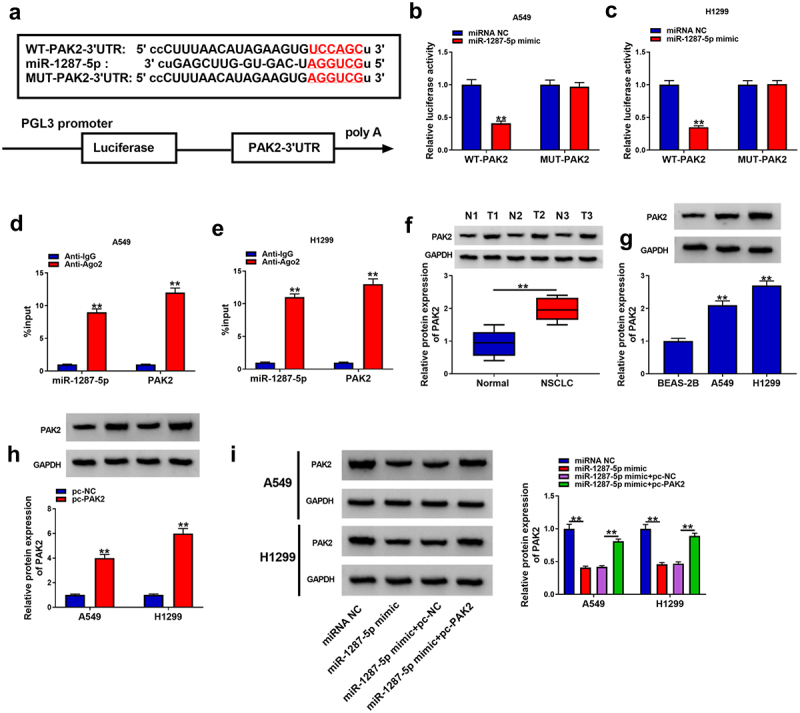
**PAK2 is a target of miR-1287-5p in NSCLC cells**. (a) The predicted binding sites of miR-1287-5p on PAK2. (b–e) Dual-luciferase reporter and RIP assays were utilized to confirm the interaction between PAK2 and miR-1287-5p in A549 and H1299 cells. (f,g) Western blot analysis of PAK2 expression in NSCLC tissues and normal adjacent tissues, as well as in normal BEAS-2B cells and NSCLC cells. (h) The transfection efficiency of pc-NC or pc-PAK2 was verified by western blot. (i) Western blot analysis was employed to investigate the impact of miR-1287-5p on PAK2 expression profile. Error bars stand for the mean ± SD of three independent measurements. *P < 0.05 and ** *P* < 0.01.

According to the starbase database, PAK2 possesses putative binding sites in miR-1287-5p (Figure 6a). Then a decline of luciferase activity in A549 and H1299 cells co-transfected with WT-PAK2 and miR-1287-5p mimic was detected (Figure 6(b,c)). At the same time, in contrast to the control IgG immunoprecipitates, miR-1287-5p and PAK2 were significantly enriched in Ago2 immunoprecipitates (Figure 6(d,e)). Thus, miR-1287-5p targeted PAK2 in NSCLC cells. Subsequently, we observed that PAK2 was elevated in NSCLC tissues and cell lines in comparison to those of control groups (Figure 6(f,g)). Additionally, pc-PAK2 transfection significantly up-regulated PAK2 in A549 and H1299 cells (Figure 6h) and also could rescue miR-1287-5p mimic-mediated PAK2 down-regulation (Figure 6i). Collectively, miR-1287-5p targeted PAK2 expression in NSCLC cells.

### 3.7 MiR-1287-5p inhibits oncogenic phenotypes in NSCLC cells via PAK2

**Figure 7.**
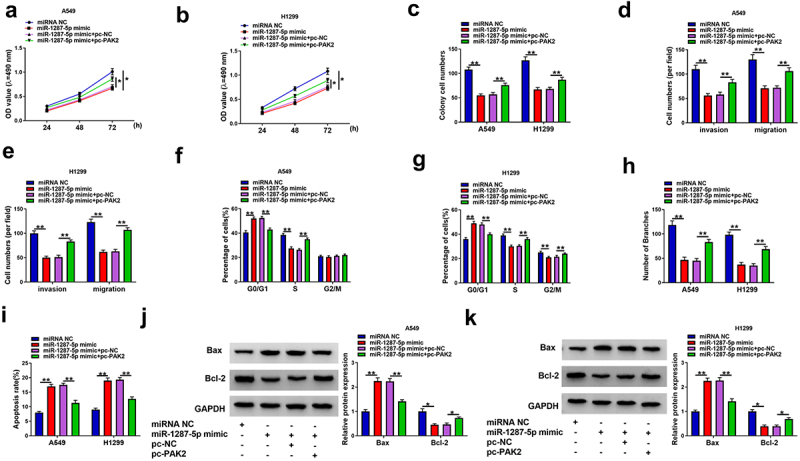
**MiR-1287-5p inhibits malignant phenotypes in NSCLC cells by targeting PAK2**. A549 and H1299 cells were transfected with miR-1287-5p and/or pc-PAK2. (a–c) MTT assay and colony formation assay for the cells proliferation; (d–e) Transwell assay for A549 and H1299 cell invasion and migration abilities. (f–g) Flow cytometry for cell cycle in A549 and H1299 cells. (h) Tube formation in HUVECs by conditioned medium from indicated A549 and H1299 cells. (i) Flow cytometry of A549 and H1299 cell apoptosis rate. (j,k) Bax and Bcl-2 protein levels in A549 and H1299 cells were detected by western blot. Error bars stand for the mean ± SD of three independent measurements. *P < 0.05 and ** *P* < 0.01.

Given the miR-1287-5p/PAK2 axis in NSCLC cells, whether PAK2 participated in the activity of miR-1287-5p in NSCLC progression was probed. We found miR-1287-5p re-expression suppressed cell proliferation (Figure 7(a–c)), migration, and invasion abilities (Figure 7(d,e)), cell cycle (Figure 7(f,g)), and tube formation (Figure 7h) and enhanced apoptosis (Figure 7(i–k)) in A549 and H1299 cells, while PAK2 overexpression attenuated these anticancer effects induced by miR-1287-5p re-expression in NSCLC cells (Figure 7(a–k)). These results implied that miR-1287-5p suppressed NSCLC progression by targeting PAK2.

### 3.8 Circ_0008717 upregulates PAK2 through binding to miR-1287-5p

**Figure 8.**
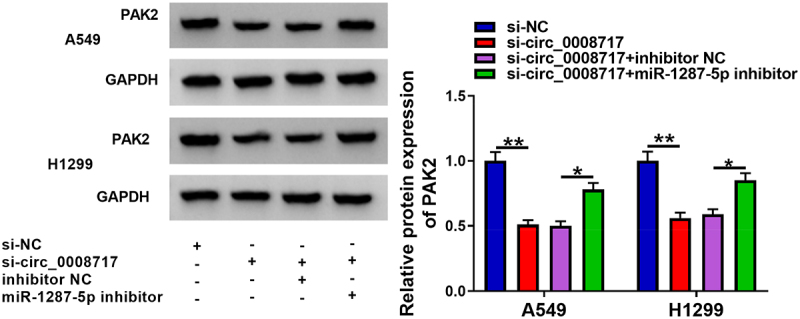
**Circ_0008717 indirectly regulates PAK2 through binding to miR-1287-5p**. After indicated transfection, the PAK2 expression in A549 and H1299 cells was detected by western blot. Error bars stand for the mean ± SD of three independent measurements. *P < 0.05 and ** *P* < 0.01.

Considering the above findings, we assumed that circ_0008717 might be able to modulate PAK2 via miR-1287-5p. As the exhibition of Figure 8a, circ_0008717 knockdown resulted in a reduction of PAK2 level, while this phenomenon was rescued by miR-1287-5p repression. Thus, we knew that circ_0008717 was able to upregulated PAK2 through absorbing miR-1287-5p.

### 3.9 *Circ_0008717 knockdown impedes tumor growth* in vivo

**Figure 9.**
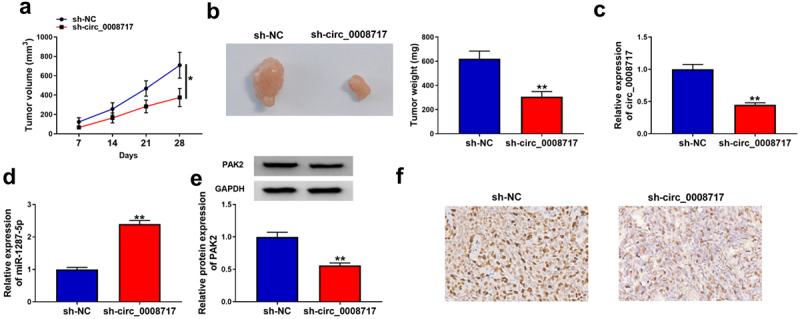
**Circ_0008717 knockdown impedes tumor growth *in vivo.*** (a,b) Tumor size and weight of xenograft were detected. (c–e) qRT-PCR or western blot was utilized to assay the levels of circ_0008717, miR-1287-5p and PAK2 in tumors from each group. (f) Representative images of PAK2 staining in the tumors. *P < 0.05 and ** *P* < 0.01.

The functions of circ_0008717 *in vivo* were further investigated through xenograft tumor models. As shown in Figure 9(a,b), tumor volume and weight were greatly inhibited in sh-circ_0008717 group compared with those in sh-NC group. Besides, molecular analysis showed that circ_0008717 level was down-regulated in tumors of sh-circ_0008717 group, suggesting successful injection of sh-circ_0008717 (Figure 9c). Meanwhile, it was observed that circ_0008717 deficiency elevated miR-1287-5p expression (Figure 9d) and reduced PAK2 expression *in vivo* (Figure 9e). Moreover, IHC assay confirmed that circ_0008717 knockdown caused decreased PAK2 expression (Figure 9f). Altogether, circ_0008717 deficiency hindered NSCLC growth *in vivo* via miR-1287-5p/PAK2 axis.

## 4 Discussion

In our study, a high expression of circ_0008717 was validated in NSCLC, circ_0008717 deficiency suppressed cell oncogenic phenotypes in NSCLC. Importantly, xenograft experiments displayed that circ_0008717 deficiency hindered NSCLC growth *in vivo*. All these data suggested the anticancer property of circ_0008717 siRNA in NSCLC. Exosomes are endosome-derived vesicles containing an abundance of cargoes and can transmit and exchange their diverse cargoes with other cells and the extracellular microenvironment, forming a complex communication network that promotes the growth and progression of cancer [20,21]. All kinds of cells can secrete exosomes, and exosomes are stable in body fluids due to their phospholipid bilayer. Moreover, they have a low-risk for cancer occurrence [22]. Thus, exosomes are considered ideal vehicles for *in vivo* delivery for therapeutic information. Importantly, it has emerged that exosomes carrying circRNAs possess therapeutic potential in tumor management [23]. For example, exosomal circRNA PDE8A released by tumor facilitated invasive growth of pancreatic cancer [7]. Exosomal circRNA_100338 enhanced the angiogenesis and invasiveness of cells to aggress HCC metastasis [19]. Exosomal circ_0006156 enhanced cell survival in papillary thyroid cancer (PTC) via miR-1178/TLR4 pathway to modulate PTC progression [24]. In this study, circ_0008717 was uncovered to be stably packaged into exosomes in NSCLC, and could be secreted into the microenvironment by exosomes. Therefore, the *in vivo* delivery of circ_0008717 siRNA via exosomes might be a promising therapeutic method for NSCLC, which still needs to be further validated in the future.

MiRNA-based therapies have been shown to hold great promise via either modulating the oncogene or the tumor suppression gene with miRNAs [10,25,26]. Based on the ceRNA hypothesis [27,28], miRNA targets of circ_0008717 in NSCLC cells were investigated. We identified and confirmed that circ_0008717 sponged miR-1287-5p in NSCLC cells. Studies showed that miR-1287-5p mediated circ_0005576-induced promotion of cervical cancer progression [29]. Besides, miR-1287-5p was decreased in breast cancer, and silencing of miR-1287-5p promoted cancer growth [30]. In NSCLC, Chang *et al*. showed miR-1287 was sponged by circ_0026134 and the oncogenic role of circ_0026134 was reversed by miR-1287 [31]. It was also identified that miR-1287 was implicated in NSCLC progression by the circ_0016760/miR-1287/GAGE1 pathway [32]. Consistent with previous research, we discovered the anticancer potential of miR-1287-5p in NSCLC. More importantly, miR-1287-5p deficiency counteracted the anticancer action of si-circ_0008717 in NSCLC progression.

PAK2 has been shown to modulate differentiation, motility, and attachment in a large number of cellular contexts, including tumor cells [33,34]. Recent studies have revealed that PAK2 was elevated in many types of cancer and performed oncogenic effects to induce malignant progression in these cancers [35–37]. Importantly, it was also shown that PAK2 reversed miR-7-5p-mediated phenotypic changes to promote NSCLC progression [38]. In our research, we validated miR-1287-5p-targeted PAK2, and overexpression of PAK2 attenuated the suppressive of miR-1287-5p in the malignant phenotypes of NSCLC cells. Moreover, we also found circ_0008717 sponged miR-1287-5p to promote PAK2 expression.

## 5 Conclusion

Above all, this study demonstrated that circ_0008717 was stably packaged into exosomes in NSCLC, and functionally, circ_0008717 was an oncogenic factor that promoted tumorigenesis in NSCLC by elevating PAK2 expression through miR-1287-5p, which might offer a novel insight into the understanding of exosomal circRNA biology in NSCLC, and the development of exosome-based therapeutic strategy for NSCLC patients.

## List of abbreviations

NSCLC non-small cell lung cancer

NTAnanoparticle tracking analysis

PAK2P21-activated kinase 2
